# Ketogenic treatment reduces the percentage of a LHON heteroplasmic mutation and increases mtDNA amount of a LHON homoplasmic mutation

**DOI:** 10.1186/s13023-019-1128-z

**Published:** 2019-06-21

**Authors:** Sonia Emperador, Ester López-Gallardo, Carmen Hernández-Ainsa, Mouna Habbane, Julio Montoya, M. Pilar Bayona-Bafaluy, Eduardo Ruiz-Pesini

**Affiliations:** 1 Departamento de Bioquímica, Biología Molecular y Celular, Universidad de Zaragoza, C/ Miguel Servet, 177. 50013, Zaragoza, Spain; 2 Instituto de Investigación Sanitaria (IIS) de Aragón, Av. San Juan Bosco, 13. 50009, Zaragoza, Spain; 3 Centro de Investigaciones Biomédicas en Red de Enfermedades Raras (CIBERER), Av. Monforte de Lemos, 3-5. Pabellon 11, Planta 0. 28029, Madrid, Spain; 4 Fundación ARAID, ARAID, Av. de Ranillas, 1-D. Planta 2º, oficina B. 50018, Zaragoza, Spain

**Keywords:** Mitochondrial DNA, Leber hereditary optic neuropathy, Ketogenic diet

## Abstract

**Background:**

The vision loss in Leber hereditary optic neuropathy patients is due to mitochondrial DNA mutations. No treatment has shown a clear-cut benefit on a clinically meaningful end-point. However, clinical evidences suggest two therapeutic approaches: the reduction of the mutation load in heteroplasmic patients or the elevation of mitochondrial DNA amount in homoplasmic patients.

**Results:**

Here we show that ketogenic treatment, in cybrid cell lines, reduces the percentage of the m.13094 T > C heteroplasmic mutation and also increases the mitochondrial DNA levels of the m.11778G > A mitochondrial genotype.

**Conclusions:**

These results suggest that ketogenic diet could be a therapeutic strategy for Leber hereditary optic neuropathy.

## Background

Leber hereditary optic neuropathy (LHON) is a kind of blindness due to retinal ganglion cells (RGC) loss provoked by pathologic mutations in the mitochondrial DNA (mtDNA), mainly in genes for respiratory complex I (CI) subunits. Three of these mutations, m.3460G > A, m.11778G > A and m.14484 T > C, account for most of LHON patients. The remaining cases are caused by a number of very rare mutations, such as m.13094 T > C [1].

The existence of mutant and wild-type mtDNA, known as heteroplasmy, is found in 10–15% of individuals [2]. Blood mutation load in these heteroplasmic individuals is directly related to the frequency of vision loss [3]. Heteroplasmy is frequent in patients who recover their vision. The lower the percentage of pathological mutation, the higher the probability of spontaneous recovery [4]. Interestingly, it was previously reported that osteosarcoma 143 cybrids with an heteroplasmic 1.9 kb mtDNA deletion grown 5 days in a medium with no glucose but with acetoacetate (AA), β-hydroxybutyrate (BHB) or both (AA + BHB) suffered a slight decrease (7–20%) in proportion of deleted mtDNA [5]. The result of this ketogenic treatment suggested a possibility to decrease the LHON pathologic point mutation load in heteroplasmic patients.

Most LHON individuals are homoplasmic, i.e. they only have mutant mtDNA, and mutation load cannot be decreased. However, not all individuals harboring a LHON homoplasmic mutation suffer the disease. In mutant homoplasmic individuals, mtDNA levels were found to be lower in patients than in healthy carriers [6]. Moreover, risk factors for LHON, whether genetic, such as mtDNA haplogroup J; physiological, such as male gender; pharmacological, for example antiretroviral therapy; or environmental, such as smoking, have also been associated with lesser mtDNA amount [7]. Remarkably, it has also been shown that neuroblastoma SH-SY5Y cybrids with an almost homoplasmic (98.6%) pathologic point mutation (m.3243A > G) showed an increase in the mtDNA copy number, with no change in the mutation load, when cultured for 28 days in a medium with ketone bodies and low glucose [8]. This result suggested that ketogenic diet (KD), a high fat and low carbohydrate diet, by increasing mtDNA levels, might also be a therapeutic strategy for LHON homoplasmic individuals.

Currently, there are no level I clinical trial data supporting the use of any medication in LHON. Education and a reduction of all the likely risk factors are the bedrock of management in LHON [9]. To explore the use of KD as a potential therapy for LHON patients, we simulated this approach in heteroplasmic and homoplasmic cybrids, trying to decrease LHON mutation load or increasing the mtDNA copy number.

## Results

### Reducing the heteroplasmic mutation percentage

**Fig. 1:**
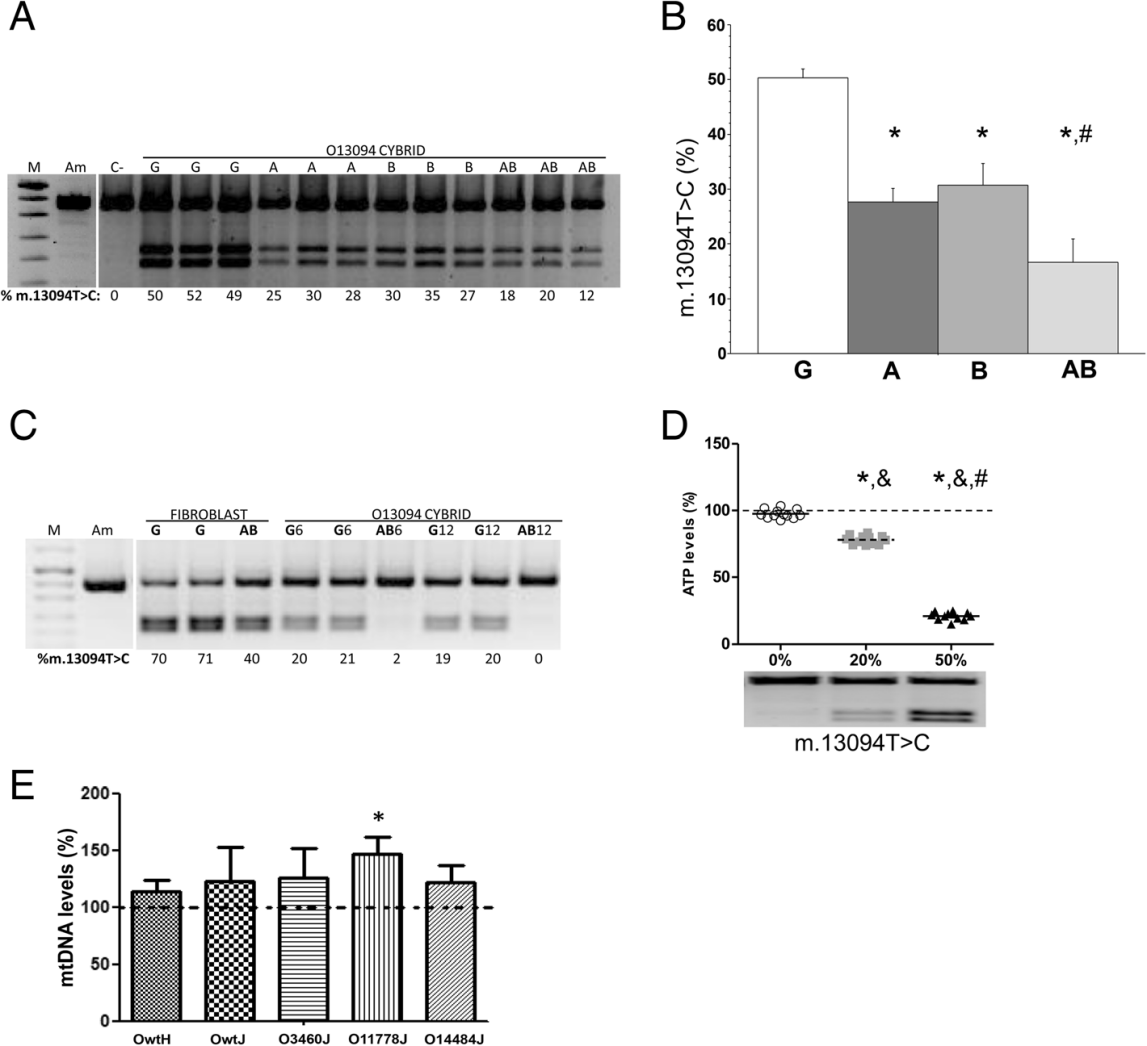
Effect of ketogenic treatment on mtDNA mutation percentage and copy number. **a** Percentage of m.13094 T > C transition. Gel showing PCR-RFLP results for cybrids with the m.13094 T > C mutation. M, molecular weight marker; Am, amplicon; C-, negative control; G, 5.5 mM glucose grown mutant cybrid; A, 5 mM acetoacetate-treated mutant cybrid; B, 5. mM β-hydroxybutyrate-treated mutant cybrid; AB, 5 mM acetoacetate + 5 mM β-hydroxybutyrate-treated mutant cybrid. Heteroplasmy produces two new bands (218 + 188 base pairs). **b** Graph showing percentage of m.13094 T > C mutation in cybrids. These percentages were obtained from the Fig. 1a gel. Statistically significant differences are indicated by * (vs G) or ^#^ (vs A or B). **c** Percentage of m.13094 T > C transition. Gel showing PCR-RFLP results for m.13094 T > C mutation, in fibroblast and cybrids, after additional ketogenic treatment. G6 and G12, cybrids with 20% mutation load grown in G medium for additional 6 and 12 days, respectively. AB6 and AB12, cybrids with 20% mutation load grown in AB medium for additional 6 and 12 days, respectively. **d** ATP levels in cybrids with different m.13094 T > C mutation load. Dashed line represents ATP levels in the Owth control cybrid. Statistically significant differences are indicated by * (vs Owth), ^&^ (vs O13094, 0%), or ^#^ (vs O13094, 20%). **e** mtDNA levels in wild-type and LHON mutant cybrids. mtDNA amount in each untreated cybrid is considered 100% (dashed line). Statistically significant differences are indicated by * (vs untreated cybrid).

In a previous work, we described a LHON patient with the m.13094 T > C mtDNA transition and confirmed its pathogenicity using cybrids [1]. These cybrids (O13094) harbored a 50.3% of the mutant allele. Here, we observed a significant decrease in the proportion of m.13094 T > C transition when cybrids were grown with no glucose in the presence of AA (up to 27.7%) or BHB (up to 30.7%) (Fig. 1a, b). Interestingly, AA + BHB further reduced the mutation load (up to 16.7%).

Moreover, when cybrids with a 20% mutation load were cultured for additional 6 or 12 days in a medium with no glucose but with AA + BHB, they practically lost the m.13094 T > C transition (Fig. 1c). A different type of cell, patient’s fibroblasts (70.5%) mutation load), also showed a reduction in the mutation load when grown in AA + BHB (up to 40%) (Fig. 1c).

To study the bioenergetic effect of the reduction in the mutation percentage, we determined the ATP levels. These levels were significantly lower in cybrids with a 50% of pathological mutation than in those with a 20% (Fig. 1d). ATP amount in both cybrids was significantly lower than that in their isogenic control, with the same mtDNA genotype except for the pathological mutation. ATP concentration was the same in the isogenic cybrid and a control cybrid from another haplogroup and with no pathological mutation. Thus, the decrease in the cybrid mutation load was associated with an increase in ATP amount to reach normal levels (Fig. 1d).

### Increasing the homoplasmic mutant mtDNA levels

We had previously observed an inverse relationship between the percentage of m.13094 T > C mutation and the mtDNA copy number [1]. The ketogenic treatment (no glucose and AA + BHB) decreased the mutation percentage but also increased mtDNA amount.

To explore the effect of ketogenic treatment on mtDNA levels of LHON homoplasmic mutant cells, we analyzed five osteosarcoma 143B cybrid cell lines: two free of pathological mutations (Owth and Owtj) and three carrying the most common LHON mutations (O3460J, O11778J and O14484J). We had previously confirmed that all three mutant cybrids are homoplasmic, and along with Owtj, share the same nuclear genetic background and belong to the same mtDNA genetic background, haplogroup J [10]. Here, we first analyzed 16 short tandem repeats (STR) and confirmed that Owth also shares the same nuclear genetic background than the previous cybrids, but it belongs to the most common European mtDNA genetic background, haplogroup H (GenBank HM103354.1).

Next, we determined their mtDNA levels after 7 days growing in no glucose, AA + BHB containing medium. Treated cybrids tent to have higher mtDNA levels (Fig. 1e). Moreover, treated O11778J cybrids showed significantly higher mtDNA levels (Fig. 1e). In osteosarcoma 143B cybrids, we previously described that mtDNA amount determines OXPHOS capacity [11].

## Discussion

The O13094 heteroplasmic cybrid, when grown in no glucose medium with AA and/or BHB, decreases its mutation load. A similar observation had been previously published [5]. The reduction in the percentage of m.13094 T > C transition may be due to a selection of healthier mitochondria or cells. In fact, it had been described a significant negative correlation between the percentage of m.13094 T > C mutation and the CI activity [12], which would explain our observation of an increase in ATP concentration when the mutation level is reduced. The catabolism of AA and BHB requires mitochondrial tricarboxylic acid cycle and oxidative phosphorylation (OXPHOS). Moreover, by reducing glucose concentration and limiting glycolysis, cells become more dependent on mitochondria to obtain energy. In fact, apparently homoplasmic SH-SY5Y cybrids reduced the m.3243A > G mutation load to 90% when its growth medium was shifted from high (25 mM) to low (2.75 mM) glucose [13]. Thus, the growing medium that we use probably selects for less mutated mitochondria or cells. It has been reported that, beside their metabolic effects, ketone bodies have also other properties, such as gene expression regulation [14]. These other properties could be responsible for the larger decrease in mtDNA mutation load when AA + BHB, instead AA or BHB, are used.

The homoplasmic cybrid O11778J significantly increases its mtDNA levels when growth in no glucose medium with AA + BHB. Although no significant, the remaining cybrids show a similar tendency. As previously commented, glucose deprivation requires a cellular energetic shift from glycolysis to OXPHOS. Cells can also grow on galactose medium but must derive much of their energy from OXPHOS [15]. In fact, aerobic oxidation of glutamine provides most of the energy when galactose is the carbohydrate in the growing medium [16]. Thus, it was reported that human cervical cancer HeLa and osteosarcoma U2OS cells increased respiration when glucose 25 mM was substituted by glucose-free/galactose 10 mM growing media [17, 18]. The higher oxygen consumption in these cells was accompanied by densely packed mitochondrial cristae, increased supercomplex activities and levels and enrichment in respiratory complex proteins [17, 18]. In HeLa cells growing in galactose medium, a non-significant increase in mtDNA levels was observed [17]. Control and LHON osteosarcoma 143B cybrids showed an increase in mtDNA amount and *MT-CO1* and *MT-ND5* mRNA levels after incubation in glucose-free/galactose 5 mM medium [19]. The reduction of glucose concentration, from 30 to 5.5 mM, also increased oxygen consumption and mtDNA copy number in HepG2 cells [20]. Human neuroblastoma SH-SY5Y cybrids showed increased oxygen consumption, CI activity, p.MT-CO2 subunit amount and mtDNA levels when glucose concentration was decreased from 25 to 2.75 mM [21]. Reduction in glucose concentration from 25 to 1 mM increased oxygen consumption in U2OS cells [18]. Human hepatocellular carcinoma HepG2 cells growing in absence of glucose showed an increase in CIV activity, mtDNA-encoded proteins and mRNAs and mtDNA amount versus cells growing at glucose 25 mM [22]. Additionally, it has been shown that KD induces mitochondrial biogenesis [23–28], frequently accompanied by increased mtDNA amount [8, 29–31]. It is important to remark that we have compared mtDNA levels in cybrids grown in medium with AA + BHB but no glucose, with those grown in glucose 5.5 mM. Therefore, the elevation in mtDNA levels when glucose concentration is reduced from 25 mM to 5.5 mM or no glucose (but with AA + BHB) probably masked a lower effect of AA + BHB on mtDNA amount. Thus, mitochondrial biogenesis and OXPHOS function, and their surrogate marker mtDNA copy number, increase with glucose deprivation, both in wild-type and mutant cells.

In mouse, a positive and significant correlation was found between mtDNA levels and uncoupled oxygen consumption in Lewis lung carcinoma LL/2-m21 cybrids [32]. In human, we had found that mtDNA copy number was lower in Western Europe haplogroup J than H osteosarcoma 143B cybrids [11]. These lower mtDNA levels were accompanied by lower mitochondrial RNA amount, significant decrease in mitochondrial protein synthesis, reduction in mitochondrial inner membrane potential and ATP levels. Moreover, mtDNA levels significantly and positively correlated with mitochondrial RNA levels, mitochondrial protein synthesis and mitochondrial inner membrane potential [11]. It was also found that mtDNA copy number was higher in East Asian macrohaplogroup M than N osteosarcoma 143B cybrids [33]. These higher mtDNA levels were accompanied by higher mitochondrial RNA amount, significant increases in respiratory complex III levels, rise in mitochondrial oxygen consumption and in NAD^+^/NADH ratio [33]. It was also reported that, after estradiol treatment, control and LHON (m.3460G > A, m.11778G > A and m.14484 T > C) mutant osteosarcoma 143B cybrids increased mtDNA copy number, oxygen consumption and mitochondrial inner membrane potential [19]. Moreover, estradiol-treated control and m.3460G > A cybrids increased *MT-CO1* and *MT-ND5* mRNA levels and p.MT-ND6 polypeptide amount [19]. Estradiol-treated control and m.11778G > A cybrids increased total ATP cellular content [19]. All these results confirm that mtDNA amount largely determines the OXPHOS function and could explain why risk factors for LHON have been associated with lesser mtDNA amount [7], and why higher mtDNA levels protect against LHON mutations, as reported in healthy homoplasmic LHON mutation carriers [6, 34]. Perhaps, mutant proteins are partially actives or, maybe, they can be found in an active/inactive dynamic equilibrium. In both cases, higher mutant protein amount would imply higher activity.

KD was used in children with epilepsy and OXPHOS defects, most of them in CI, the one affected in LHON patients. This diet was a safe and effective therapy for these patients [35]. KD was applied to a young girl suffering Alpers-Huttenlocher syndrome due to a pathological mutation in the mtDNA polymerase gamma. This syndrome causes mtDNA depletion and defective OXPHOS function. She clinically improved [36]. KD has been also applied to two patients with mtDNA point mutation, provoking clinical improvement [37, 38]. However, KD effects on mitochondria were not analyzed in any of these patients. On the other hand, KD has not been used in LHON patients, but LHON patients suffer from RGC loss and, in rodent models of RGC damage, it was shown that KD have a RGC neuroprotective effect, preserving its structure and function, increasing mitochondrial respiration and up-regulating mitochondrial biogenesis [39, 40].

## Conclusions

Mitochondrial biogenesis is a potential therapeutic target for LHON [7, 41], and our results suggest that KD might be effective for heteroplasmic and homoplasmic LHON patients.

## Methods

Cybrids were generated by fusing osteosarcoma 143B cells with mitochondria but no mtDNA, rho^0^ cells, to platelets, with mitochondria and mtDNA but no nucleus or nuclear DNA (nDNA), from three homoplasmic (m.3460G > A, m.11778G > A and m.14484 T > C) LHON patients, one heteroplasmic (m.13094 T > C) LHON patient and two control individuals, according to previously described protocols [42]. These cells should share the nDNA and the growing conditions and should differ in their mtDNA genotype. Institutional review boards of all participating centers approved this study (CEICA CP-12/2014). Informed consent was obtained from all subjects.

Growing media were DMEM supplemented with 10 mM HEPES, 4 mM L-glutamine, 1 mM sodium pyruvate, 10% fetal bovine serum (FBS) and 25 mM (high glucose- HG); 5.5 mM (low glucose- LG); or no glucose (NG). In KD, fatty acids are used in liver to produce ketone bodies, mainly AA and BHB. Cybrids and fibroblasts were defrosted and grown in HG medium during 3 days and passed to LG medium for another 2 days. Then, we seeded 1 × 10^6^ cells from each cell line in 100 mm-plates with LG medium. Next day, the medium was changed to LG medium plus 50 μg/ml uridine or NG medium plus 50 μg/ml uridine and 5 mM AA and/or 5 mM BHB. Cells were cultured for 7 days, changing the culture medium every second day, and no allowing them to overcome a confluence of 80%. These AA and BHB concentrations were selected in agreement with physiological levels in patients on KD [5]. Cells were kept in 5% CO_2_ at 37 °C.

Total DNA was extracted using a commercial kit. The confirmation of LHON mutations was performed by polymerase chain reaction/restriction fragment length polymorphism (PCR/RFLP), as previously reported [1], by using specific oligonucleotides primers that corresponded to each primary mutation (m.3460G > A/MT-ND1, m.11778G > A/MT-ND4 and m.14484 T > C/MTND6). The percentage of m.13094 T > C mutation was also analyzed by PCR/RFLP by using primers 12906Fw (5′-CCTACACTCCAACTCATGAGACCCA-3′) and 13310Rv (5′-TGCTAGGTGTGGTTGGTTGATGCCG − 3′). The amplicon size is 406 base pairs (bp), and the PCR conditions 95 °C 5 min (95 °C 45 s / 64 °C 30 s / 72 °C 2 min) 35 cycles, 72 °C 5 min. The restriction enzyme AluI cuts the mutant sequence in two 218 + 188 bp fragments [12]. The mutation percentages were obtained with the GelProAnalyzer 4.0 program by scanning bands from RFLP gels. The mtDNA copy number was determined by the qRT-PCR method, as described elsewhere [43]. Briefly, a 123 bp (807 to 929) fragment of mitochondrial 12S RNA gene was analyzed. The primers used to detect the mtDNA 12S sequences were MT-L (5′-CCACGGGAAACAGCAGTGATT-3′) and MT-H (5′-CTATTGACTTGGGTTAATCGTGTGA-3′) and were used together with the mtDNA specific fluorescent-type MGB (minor groove binding) Taqman probe, which was labeled internally by the fluorescent dye FAM (5′-TGCCAGCCACCGCG-3′). Probe and primers designs were implemented with Primer Express 2.0 software. The mtDNA quantity was corrected by simultaneous measurement of a single copy nuclear RNase P gene. To quantify nDNA, a commercial kit was used (PDARs RNAseP), and the nDNA specific fluorescent probe was labeled internally using the fluorescent dye VIC.

ATP amount, normalized by the cell number, was measured following previously described protocols with slight modifications [44], using the CellTiter-Glow Luminiscent Cell Viability Assay according to the manufacturer’s instructions. Briefly, 10,000 cells/well were seeded 14–16 h before measurement. Then, cells were washed twice with PBS and incubated for 2 h in record solution with 5 mM 2-deoxy-D-glucose plus 1 mM pyruvate (oxidative ATP production). Cells were lysed, and lysates were incubated with the luciferin/luciferase reagents. Samples were measured using a microplate luminometer, and the results referred to cell number.

Data for mean and standard deviation are presented. One-way ANOVA, Bonferroni post-hoc test and t-test were used to compare parameters. *P*-values lower than 0.05 were considered statistically significant.

## Data Availability

The datasets used and/or analysed during the current study are available from the corresponding author on reasonable request.
